# A Sentinel-2 derived dataset of forest disturbances occurred in Italy between 2017 and 2020

**DOI:** 10.1016/j.dib.2022.108297

**Published:** 2022-05-21

**Authors:** Saverio Francini, Gherardo Chirici

**Affiliations:** a Department of Agriculture, Food, Environment and Forestry, Università degli Studi di Firenze, Via San Bonaventura, 13, 50145 Firenze, Italy; b Fondazione per il Futuro delle Città, Firenze, Italy

**Keywords:** Google Earth Engine, Remote Sensing, Open-access, Big data, Cloud computing, forest fires, wind damages, forest harvestings

## Abstract

Forests absorb 30% of human emissions associated with fossil fuel burning. For this reason, forest disturbances monitoring is needed for assessing greenhouse gas balance. However, in several countries, the information regarding the spatio-temporal distribution of forest disturbances is missing. Remote sensing data and the new Sentinel-2 satellite missions, in particular, represent a game-changer in this topic.

Here we provide a spatially explicit dataset (10-meters resolution) of Italian forest disturbances and magnitude from 2017 to 2020 constructed using Sentinel-2 level-1C imagery and exploiting the Google Earth Engine GEE implementation of the 3I3D algorithm. For each year between 2017 and 2020, we provide three datasets: (i) a magnitude of the change map (between 0 and 255), (ii) a categorical map of forest disturbances, and (iii) a categorical map obtained by stratification of the previous maps that can be used to estimate the areas of several different forest disturbances. The data we provide represent the state-of-the-art for Mediterranean ecosystems in terms of omission and commission errors, they support greenhouse gas balance, forest sustainability assessment, and decision-makers forest managing, they help forest companies to monitor forest harvestings activity over space and time, and, supported by reference data, can be used to obtain the national estimates of forest harvestings and disturbances that Italy is called upon to provide.

## Specifications Table

**Table:** 

Subject	*Forestry*
Specific subject area	*Remote Sensing and forest change detection*
Type of data	Image
How the data were acquired	Computed in and exported from Google Earth Engine
Data format	Raw Analyzed Filtered
Description of data collection	Data queried, analysed, and processed in Google Earth Engine from L1C Sentinel 2A and 2B satellites. Cloud coverage is limited to 40%. Time windows to filter data is May-20 to Sep-10 of years from 2016 to 2021
Data source location	Data covering all of the administrative regions of Italy. Spatial extent (WGS 84, EPSG: 4326): Upper Left: 6.6239074105121158, 36.6491076353945857 Lower Right: 18.5144576687601656,47.0946279276079736
Data accessibility	Repository name: Mendeley Data Data identification number: 10.17632/5jyp57ymw4.1 Direct URL to data: https://data.mendeley.com/datasets/5jyp57ymw4/1
Related research article	S. Francini, R.E. McRoberts, G. D'Amico, N.C. Coops, T. Hermosilla, J.C. White, M.A. Wulder, M. Marchetti, G. Scarascia Mugnozza, G. Chirici, An open science-open data approach for statistically robust estimation of forest disturbance area, International Journal of Applied Earth Observation and Geoinformation 106 (2022) https://doi.org/10.1016/j.jag.2021.102663

## Value of the Data

• The spatio-temporal distribution of forest disturbances is unknown in several countries including Italy, but this information is essential for estimating the greenhouse gas balance and the sustainability of forest management.
• The maps we provide are obtained using Sentinel-2 images (10 meters resolution) and developed using an innovative algorithm specifically developed for Mediterranean ecosystems.
• Any researchers or remote sensing scientists may benefit from this data. The data we provide are easy-to-use and they can be used to obtain area estimates of different forest disturbances. Plus, researchers of different fields may provide more wide understanding and insights of the maps we provide.
• This data can help decision-makers to support forest harvestings activity management and help forest companies to monitor forests over space and time.
• These data allow monitoring of extreme disturbance events (wind damages, forest fires, drought phenomena, insect outbreaks) over time and space. Due to climate change, this kind of monitoring is more essential than ever.
• If supported by reference data, the maps we provide can be used to obtain estimates of forest disturbances that Italy and other countries are called upon to provide.

## 1 Data Description

**Fig. 1:**
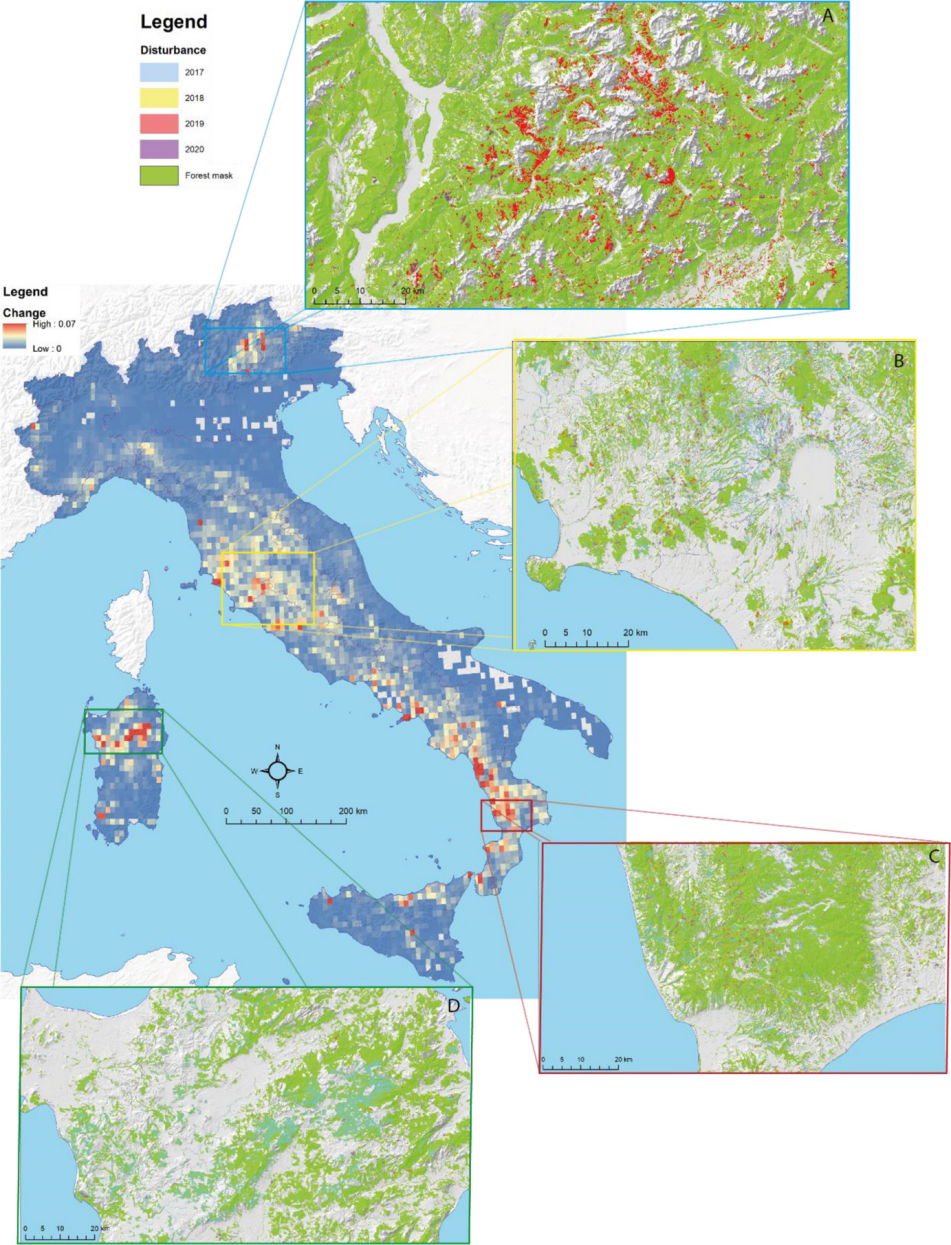
Forest disturbances predicted in Italy between 2017 and 2020 using the 3I3D algorithm. The percentage of the forests that were disturbed over Italy considering the whole period is shown in the largest panel using a pixel size of 1-km. The four smaller panels (a-d) show zooms of the disturbance boolean maps.

The dataset provides information on Italian forest disturbances between August 2016 and August 2020 (Fig. 1), has a spatial resolution of 10 meters, and consists of a 12-TIF-images collection, three images for each year from 2017 to 2020: (i) the *disturbance magnitude map*, the *disturbance boolean map,* and (iii) the *disturbance buffer map* [1]. The *disturbance magnitude map* assumes values between 0 and 255 and indicates for each forested pixel the magnitude of the disturbance. No-forest pixels have no data in this map. The *disturbance boolean map* is a categorical map created applying a threshold (224, [2]) to the *disturbance magnitude map* and classifies Italy into (1) forest disturbed, (2) forest undisturbed, and (255) non-forest. The *disturbance buffer map* is a categorical map obtained by augmenting the *disturbance boolean map* with an additional *buffer* class consisting of a two-pixels buffer (20 m) on each side of the disturbance boundary. It classifies Italy into (1) forest disturbed, (2) forest undisturbed, (3) buffer, and (255) no-forest. This image can be used to produce forest disturbance area estimates following the procedure we presented in Francini et al., (2022) and using the R package we provide on GitHub (https://github.com/saveriofrancini/AreaEstimator3I3DGEE).

Data are stored as 8-bit unsigned integers and compressed values (deflate, predictors = 2). This results in a dataset covering the entire of Italy and consisting of 12 images TIF files with a size from 325 megabytes to 1.3 gigabytes (5.85 gigabytes in total), a very small memory requirement considering that a single Sentinel-2 image (110 km per 110 km) has a size of two gigabytes. This makes the Italian 3I3D forest disturbance dataset easy to store, manage, and work with.

## 2 Experimental Design, Materials and Methods

The forest disturbance data we provide was computed using the 3I3D algorithm [1,2] implemented in Google Earth Engine GEE, a cloud computing platform that combines a multi-petabyte catalog of satellite imagery and geospatial datasets with planetary-scale analysis capabilities [3]. To map forest disturbances for a specific year, 3I3D uses three Sentinel-2 cloud-free composites: (1) one from the summer of the year before the disturbance; (2) one from the summer of the year of interest, when the disturbance happened; and (3) one from the summer of year after the disturbance. For these three years, all Sentinel-2 images with less than 40% cloud cover and acquired over Italy between May 20 and September 10 are selected. Clouds and cirrus are masked out from each image using the Sentinel-2 clouds probability dataset (https://developers.google.com/earth-engine/datasets/catalog/COPERNICUS_S2_CLOUD_PROBABILITY) which – by using a gradient boost base algorithm (https://github.com/microsoft/LightGBM) - indicates between 0% and 100% the probability that each pixel is covered by clouds. As suggested by the data provider, we masked out all pixels having a probability to be cloudy greater than 65%. For pixels with multiple observations acquired on different dates, we applied the Medoid process presented by [4] and implemented by [1] which allowed us to obtain six (2016-2021) cloud-free image composites. For each year between 2017 and 2020, a forest disturbances map was created using 3I3D [1], which analysed the trends over the three consecutive years for the three vegetation indices (3I) used as the axes of three-dimensional feature space (3D). The three indices are (i) the Normalized Difference Moisture Index NDMI [5], (ii) the Normalized Burn Ratio NBR [6], and (iii) the Moisture Stress Index MSI [7]. Studying the 3D changes occurring in the 3I, 3I3D calculates a *magnitude map*: pixels with a *magnitude* greater than 88% (a 224 threshold in a 0-255 scale) are classified as forest disturbance ([2] – equations 4-6 and paragraph 2.3.1.) and stored in the *disturbance boolean map*. No-forest areas were excluded from the analysis using a detailed and accurate (overall accuracy of 91%  – [8]) forest mask of Italy [9]. Disturbances smaller than 0.1 ha are removed [10]. Finally, the *buffer map* is constructed by augmenting the *boolean map* with an additional *buffer* class consisting of a two-pixel buffer (20 m) on each side of the disturbance boundary. To have details on why the buffer map class was constructed, see [1] where we provide a procedure to obtain forest disturbance area estimates by integrating the *buffer map* with reference data. JavaScript codes of the whole procedure are open access (https://code.earthengine.google.com/?accept_repo=users/sfrancini/S23I3D).

## CRediT Author Statement

**Francini Saverio** and **Gherardo Chirici:** Conceptualization, Methodology, Software, Writing – review & editing; **Gherardo Chirici:** Visualization; **Saverio Francini** Original draft.

## Declaration of Competing Interest

The authors declare that they have no known competing financial interests or personal relationships that could have appeared to influence the work reported in this paper.

## Data Availability

A Sentinel-2 derived dataset of forest disturbance occurred in Italy between 2017 and 2020 (Original data) (Mendeley Data).
